# Non-invasive Standardised Uptake Value for Verification of the Use of Previously Validated Reference Region for [^18^F]Flortaucipir and [^18^F]Florbetapir Brain PET Studies

**DOI:** 10.1007/s11307-020-01572-y

**Published:** 2021-01-14

**Authors:** Bart M. de Vries, Tessa Timmers, Emma E. Wolters, Rik Ossenkoppele, Sander C. J. Verfaillie, Robert C. Schuit, Philip Scheltens, Wiesje M. van der Flier, Albert D. Windhorst, Bart N. M. van Berckel, Ronald Boellaard, Sandeep S. V. Golla

**Affiliations:** 1 Radiology and Nuclear Medicine, Amsterdam UMC, Vrije Universiteit Amsterdam, De Boelelaan 1117, Amsterdam, The Netherlands; 2 Alzheimer Center and Department of Neurology, Amsterdam UMC, Vrije Universiteit Amsterdam, De Boelelaan 1117, Amsterdam, The Netherlands; 3 Epidemiology & Biostatistics, Amsterdam UMC, Vrije Universiteit Amsterdam, De Boelelaan 1117, Amsterdam, The Netherlands

**Keywords:** Standardised uptake value, [^18^F]Flortaucipir, [^18^F]Florbetapir, SRTM, Alzheimer’s disease

## Abstract

**Purpose:**

The simplified reference tissue model (SRTM) is commonly applied for the quantification of brain positron emission tomography (PET) studies, particularly because it avoids arterial cannulation. SRTM requires a validated reference region which is obtained by baseline-blocking or displacement studies. Once a reference region is validated, the use should be verified for each new subject. This verification normally requires volume of distribution (*V*_T_) of a reference region. However, performing dynamic scanning and arterial sampling is not always possible, specifically in elderly subjects and in advanced disease stages. The aim of this study was to investigate the use of non-invasive standardised uptake value (SUV) approaches, in comparison to *V*_T_, as a verification of the previously validated grey matter cerebellum reference region for [^18^F]flortaucipir and [^18^F]florbetapir PET imaging in Alzheimer’s disease (AD) patients and controls.

**Procedures:**

Dynamic 130-min [^18^F]flortaucipir PET scans obtained from nineteen subjects (10 AD patients) and 90-min [^18^F]florbetapir dynamic scans obtained from fourteen subjects (8 AD patients) were included. Regional *V*_T_’s were estimated for both tracers and were considered the standard verification of the previously validated reference region. Non-invasive SUVs corrected for body weight (SUV_BW_), lean body mass (SUL), and body surface area (SUV_BSA_) were obtained by using later time intervals of the dynamic scans. Simulations were also performed to assess the effect of flow and specific binding (BP_ND_) on the SUVs.

**Results:**

A low SUV corresponded well with a low *V*_T_ for both [^18^F]flortaucipir and [^18^F]florbetapir. Simulation confirmed that SUVs were only slightly affected by flow changes and that increases in SUV were predominantly determined by the presence of specific binding.

**Conclusions:**

In situations where dynamic scanning and arterial sampling is not possible, a low SUV_(80–100 min)_ for [^18^F]flortaucipir and a low SUV_(50–70 min)_ for [^18^F]florbetapir may be used as indication for absence of specific binding in the grey matter cerebellum reference region.

**Supplementary Information:**

The online version contains supplementary material available at 10.1007/s11307-020-01572-y.

## Introduction

In quantitative dynamic brain PET studies, the simplified reference tissue model (SRTM) is one of the most frequently used models, particularly because it avoids arterial cannulation and metabolite measurements [1, 2]. However, the use of SRTM requires a validated reference region. Validation of the reference region is done using blocking or displacement studies along with histopathological assessments on post-mortem tissue samples. Due to expenses, complex study design, and procedures, the quality control (QC) of the previously validated reference region is currently done by comparing whether the distribution volume (*V*_T_) of the previously validated reference region remains unaffected regardless of healthy or Alzheimer’s disease (AD) subjects. Absence of *V*_T_ differences between the investigated subject groups suggests that the previously validated reference region has no or similar specific binding and therefore is verified to be used as the reference region. Estimation of the *V*_T_ requires a metabolite-corrected plasma input function (IF). Obtaining an IF is thus important for quantification, but requires a measurement of the arterial blood activity concentration, which in turn requires arterial cannulation. Arterial sampling and dynamic scanning is, however, not always possible in poorly conditioned patient groups, *e.g.* AD patients [3]. A non-invasive approach such as the standardised uptake value (SUV) would thus be desirable for QC of the previously validated reference region in situations where dynamic scanning and arterial sampling is not feasible. SUV metrics are already commonly used in clinical oncology imaging [4]. It ideally removes variability introduced by differences in patient size and amount of injected tracer [4, 5], and therefore is not influenced by differences in injected doses and population.

Depositions of amyloid beta (Aβ) and tau are one of the core pathological hallmarks of AD [6–10]. Aβ and tau accumulation can be visualised using [^18^F]florbetapir [11] and [^18^F]flortaucipir [12] PET. These Aβ and tau depositions are generally not evident in the grey matter cerebellum and have been validated as the reference region for both tracers [13–15].

Ideally, the use of the grey matter cerebellum as the reference region must be verified again for every new subject and cohort. This is important since the grey matter cerebellum could be affected in situations such as advanced AD pathology and therefore will have increased specific binding. This results in the cerebellum grey matter as an unsuitable reference region and would lead to unreliable quantification [16]. In a group-level study, the subject should be removed from the analysis. However, as stated earlier, it is difficult or not feasible to obtain dynamic scanning along with arterial sampling. Moreover, in a clinical setup, static scanning is usually preferred. Henceforth, a simpler QC process where dynamic scanning and arterial sampling is not necessary to verify the use of the grey matter cerebellum is required for these kinds of tracer studies.

Although several studies have been performed in line with finding an ideal reference region either by standardisation using centiloid scoring or by head on performance comparisons of different possible reference regions, it is still an open question [17–20]. So, the focus of this study was not to validate an ideal reference region; instead, the aim was to determine whether non-invasive SUV approaches could be used for verification (not validation) of the grey matter cerebellum as the reference region for individual scans in case of [^18^F]florbetapir and [^18^F]flortaucipir PET studies.

## Materials and Methods

### Participants

In this study, [^18^F]flortaucipir PET scans derived from nineteen subjects (10 AD patients and 9 controls) [12, 21] and [^18^F]florbetapir dynamic scans derived from fourteen subjects (8 AD patients and 6 controls) [11] were included. Other patient demographics can be found in previous studies from Golla et al. [11, 12, 21]. Before enrolment, all subjects provided written informed consent and the studies were approved by the Medical Ethics Review Committee of Amsterdam UMC, location VUMC.

### Data Acquisition

The [^18^F]flortaucipir PET scans were performed using a Gemini TF-64 PET/CT scanner (Philips Medical Systems, Best, The Netherlands), and the [^18^F]florbetapir PET scans were obtained using an Ingenuity TF PET/CT scanner (Philips Medical Systems, Best, The Netherlands). All subjects received a venous cannulation for injecting the tracer and a radial artery cannulation for arterial sampling. Head movements were limited by placing a head holder and with frequent monitoring during the scan with laser beams and facial marks. Subjects received a low-dose CT for attenuation correction, followed by an intravenous (IV) bolus injection of 225 ± 16 MBq [^18^F]flortaucipir or a IV bolus injection of 294 ± 32 MBq [^18^F]florbetapir.

Following the [^18^F]flortaucipir injection, a 130-min dynamic emission scan was obtained (60-min acquisition, 20-min break, followed by a 50-min acquisition). Before the second PET session of 50 min, a second low-dose CT scan was acquired. The first PET session was divided into 19 frames (1 × 15, 3 × 5, 3 × 10, 4 × 60, 2 × 150, 2 × 300, 4 × 600), and the second PET session was divided into 10 frames of 300 s. During the first PET session, the radioactivity concentration in arterial blood was assessed *via* continuous arterial sampling and manual arterial blood samples were taken at 5, 10, 15, 20, 40, 60, 80, 105, and 130 min post injection for metabolite analysis.

For [^18^F]florbetapir, a 90-min dynamic emission scan was performed. The radioactivity concentration in arterial blood was assessed *via* continuous arterial sampling, and manual arterial blood samples were taken at 5, 10, 20, 40, 60, 75, and 90 min post injection for metabolite analysis.

T1-weighted MR images for structural information were acquired using a 3.0-T Ingenuity TF PET/MR (Philips Medical Systems, Cleveland, OH, USA) for the [^18^F]flortaucipir study and a Signa HDxt MRI (General Electric, Milwaukee, WI, USA) scanner for the [^18^F]florbetapir study.

### Data Analysis

The T1-weighted MR images were co-registered to PET [22]. Regions of interests (ROIs) were delineated on the co-registered MRI scans using PVElab [23] and Hammers’ template [24], and subsequently, time activity curves (TACs) were generated. As evaluated and described by Golla et al., ROI TACs (grey matter) were fitted and the regional *V*_T_’s were estimated using a reversible two-tissue compartment model (2T4K_V_B_) for both [^18^F]flortaucipir [12] and [^18^F]florbetapir [11]. The regional *V*_T_’s were considered the gold standard for verification of the previously validated grey matter reference region. The SUV metrics corrected for body weight (SUV_BW_ (Eq. 1)), lean body mass (LBM) (SUL (Eq. 2)), and body surface area (BSA) (SUV_BSA_ (Eq. 3)) were calculated by correcting the average activity of the ROIs for either one or more of the demographic information (patient body weight (kg), patient length (cm)) and injected doses (MBq).

1 $$ {\mathrm{SUV}}_{\mathrm{BW}}(t)=\raisebox{1ex}{${C}_{\mathrm{PET}}(t)$}\!\left/ \!\raisebox{-1ex}{$\left(\mathrm{Injected}\ \mathrm{Radioactivity}/\mathrm{Weight}\right)$}\right. $$

2 $$ \mathrm{SUL}(t)=\raisebox{1ex}{${C}_{\mathrm{PET}}(t)$}\!\left/ \!\raisebox{-1ex}{$\left(\mathrm{Injected}\ \mathrm{Radioactivity}/\mathrm{LBM}\right)$}\right. $$

3 $$ {\mathrm{SUV}}_{\mathrm{BSA}}(t)=\raisebox{1ex}{${C}_{\mathrm{PET}}(t)$}\!\left/ \!\raisebox{-1ex}{$\left(\mathrm{Injected}\ \mathrm{Radioactivity}/\mathrm{BSA}\right)$}\right. $$

LBM using body weight and body mass index (BMI) was estimated using Eqs. 4 and 5. BSA using body weight and length was estimated using Eq. 6.

4 $$ \mathrm{LBM}\ \mathrm{male}=9.27\ast \frac{\mathrm{Weight}}{6.68+216\ast \mathrm{BMI}} $$

5 $$ \mathrm{LBM}\ \mathrm{female}=9.27\ast \frac{\mathrm{Weight}}{8.78+244\ast \mathrm{BMI}} $$

6 $$ \mathrm{BSA}=0.007184\ast {\mathrm{Weight}}^{0.425}\ast {\mathrm{Length}}^{0.725} $$

SUV metrics for ROIs were obtained by averaging the *C*_PET_ (*t*) over 80–100 min and 50–70 min for [^18^F]flortaucipir and [^18^F]florbetapir, respectively, as they are internationally accepted SUV intervals [11, 12, 18].

In this study, tau- and Aβ-specific regions, as shown in Supplementary Table 1 (see ESM), were defined using a *V*_T_ threshold of respectively 8.5 and 5.5, and this threshold was based upon the highest *V*_T_ value that was observed in healthy controls. The regions with *V*_T_ higher than the threshold were assumed to have specific tau or Aβ signal, and the regions with *V*_T_ lower than the threshold were assumed regions without specific tau or Aβ signal. Evaluations of the performance of the SUVs and identification of the optimal SUV metric(s) in this regard were performed by comparing the SUV regional values from the tau-/Aβ-specific regions and grey matter cerebellum to the respective *V*_T_’s. Thereafter, optimal SUV(s) for regions without specific binding should first be assessed, to obtain a SUV range (SUV range [$-2\mathrm{SD}\le \overline{\mathrm{SUV}}\le +2\mathrm{SD}$] from the grey matter cerebellum region in AD patients and healthy controls). Once these ranges for the SUVs are known, they can then be used to verify the use of the previously validated grey matter cerebellum reference region in new a subject or subject groups for which arterial sampling is not feasible. In other words, the SUV range can be used to confirm absence of specific binding in the reference region in case the specific SUV value is in between the established range.

### Statistical Analysis

Correlation (Pearson) analyses were performed between the SUVs and *V*_T_ to evaluate the performance of the SUVs for verification of the reference region, in particular to explore if a low SUV would correspond to a low *V*_T_. To investigate regional (Hammers’ ROIs) correspondence between the SUVs and *V*_T_, scatter plots for both AD patients and controls were obtained. *T* tests were used to assess the significance for all comparisons. Subsequently, box and whisker plots were used to visualise the correspondence of the SUVs to the *V*_T_ to verify the grey matter cerebellum as the reference region. The performance of the metrics to differentiate between the tau- or Aβ-specific regions and cerebellum grey matter was also visualised with the box and whisker plots.

### Flow and Binding Potential Simulations

Effect of flow and specific binding on the SUVs was also examined using simulations for [^18^F]flortaucipir and [^18^F]florbetapir, respectively. Simulated noise-free time activity curves (TACs) mimicking AD tracer kinetics (2T4K_V_B_) with effect of either flow (*K*_1_ and *k*_2_: ± 25 % in steps of 5 %) or specific binding (BP_ND_: ± 25 % in steps of 5 %) were obtained. Simulated TACs represented tau-specific (temporal gyri) and Aβ-specific (whole-brain grey matter) region kinetics, and also reference region (cerebellum grey matter) kinetics. In Table 1, the used settings are indicated. These settings were derived from the regional kinetic micro-/macro-parameters estimated from the plasma input modelling of the available [^18^F]florbetapir and [^18^F]flortaucipir scans. Subsequently, the effect of flow or specific binding on the SUVs was calculated using Eq. 7.

7 $$ \mathrm{Flow}\ \mathrm{or}\ {\mathrm{BP}}_{\mathrm{ND}}\ \mathrm{effect}=1-\raisebox{1ex}{$\Big({\mathrm{SUV}}_{\mathrm{simulated}}$}\!\left/ \!\raisebox{-1ex}{${\mathrm{SUV}}_{\mathrm{reference}}\Big)$}\right. $$

**Table 1.** Settings of the simulations

	**Temporal gyri (tau-specific region)**	**Whole cerebellum (reference region)**
**[**^**18**^**F]flortaucipir**	**Reversible two-tissue compartment model**	**Reversible single-tissue compartment model**
*K*_1_	0.26659	0.34719
*k*_2_	0.037995	0.058382
*k*_3_	0.014963	-
BP_ND_	0.37417	-
*V*_B_	0.097315	0.12744
	**Whole-brain grey matter (Aβ-specific region)**	**Whole cerebellum (reference region)**
**[**^**18**^**F]florbetapir**	**Reversible two-tissue compartment model**	**Reversible two-tissue compartment model**
*K*_1_	0.32162	0.35122
*k*_2_	0.10789	0.1305
*k*_3_	0.028032	0.016103
BP_ND_	1.0324	0.79399
*V*_B_	0.08521	0.081217

## Results

**Fig. 1.**
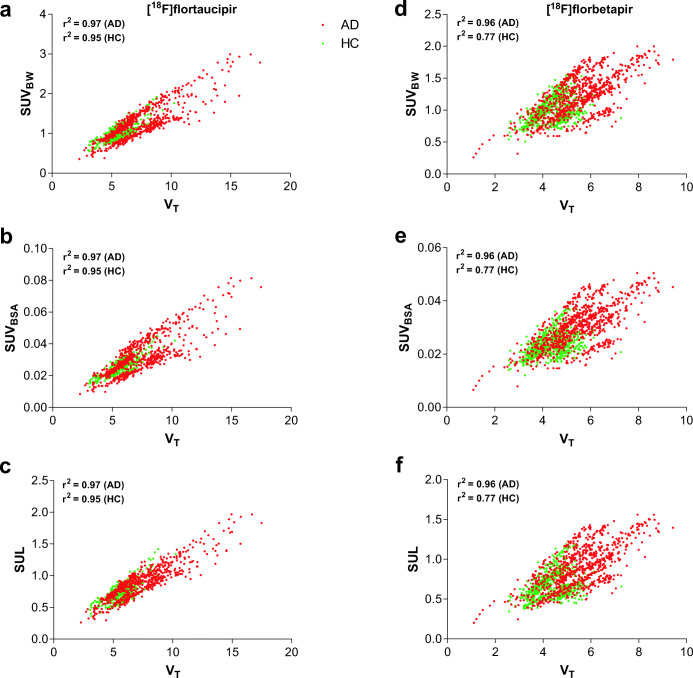
Scatter plots between SUVs and *V*_T_ for both AD patients (red) and controls (green) for all the regions of interest (tau/Aβ-specific regions, healthy regions, and grey matter cerebellum). Supplementary correlation coefficients for both AD patients and controls are also included.

Demographic and clinical data of the subjects for both [^18^F]flortaucipir and [^18^F]florbetapir are shown in Table 2. No significant differences (*p* < 0.05) were observed between the subject groups’ demographics for each tracer. Fig. 1 illustrates the correlation between the SUVs and the *V*_T_ for both [^18^F]flortaucipir and [^18^F]florbetapir for all Hammers’ template ROIs. For [^18^F]flortaucipir, a strong positive correlation was found between the SUVs and *V*_T_ for both the AD patients (*r*^2^ = 0.97) and the controls (*r*^2^ = 0.95). For [^18^F]florbetapir, a high positive correlation between *V*_T_ and all the SUVs was observed for the AD patients (*r*^2^ = 0.96), whereas for the controls, a bit lower positive correlation was found (*r*^2^ = 0.77). In addition, the regression slopes between the *V*_T_ and SUVs appeared to be subject dependent, predominantly in case of [^18^F]florbetapir.

**Table 2.** Demographic and clinical characteristics of the subjects

	AD patients	Controls
[^18^F]flortaucipir		
*n*	10	9
Age (years ± SD)	64.4 ± 7.2	69.8 ± 4.5
MMSE ± SD	23.9 ± 3.1	29.1 ± 0.6
Weight (kg ± SD)	76.3 ± 10.7	82.4 ± 13.6
Length (cm ± SD)	175.4 ± 7.5	180.0 ± 7.6
[^18^F]florbetapir		
*n*	8	6
Age (years ± SD)	65.6 ± 5.7	64.8 ± 3.3
MMSE ± SD	21.3 ± 2.3	29.8 ± 0.4
Weight (kg ± SD)	83.6 ± 12.4	86.2 ± 16.0
Length (cm ± SD)	180.3 ± 7.2	176.0 ± 14.6

**Fig. 2.**
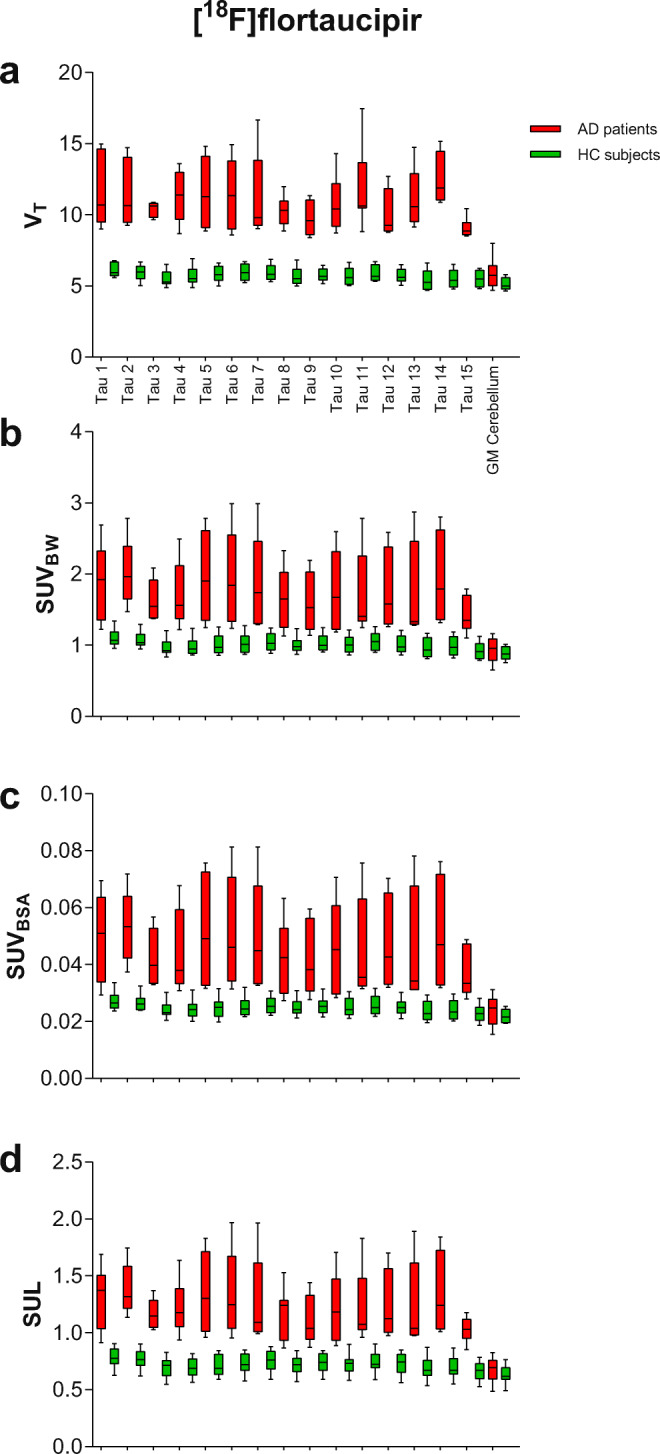
Boxplots for the SUVs and the *V*_T_ for the tau-specific regions (*V*_T_ higher than 5.5) and grey matter cerebellum of [^18^F]flortaucipir for both AD patients (red) and controls (green). Names of the tau-specific regions used in this figure are illustrated in Supplementary Table 1.

**Fig. 3.**
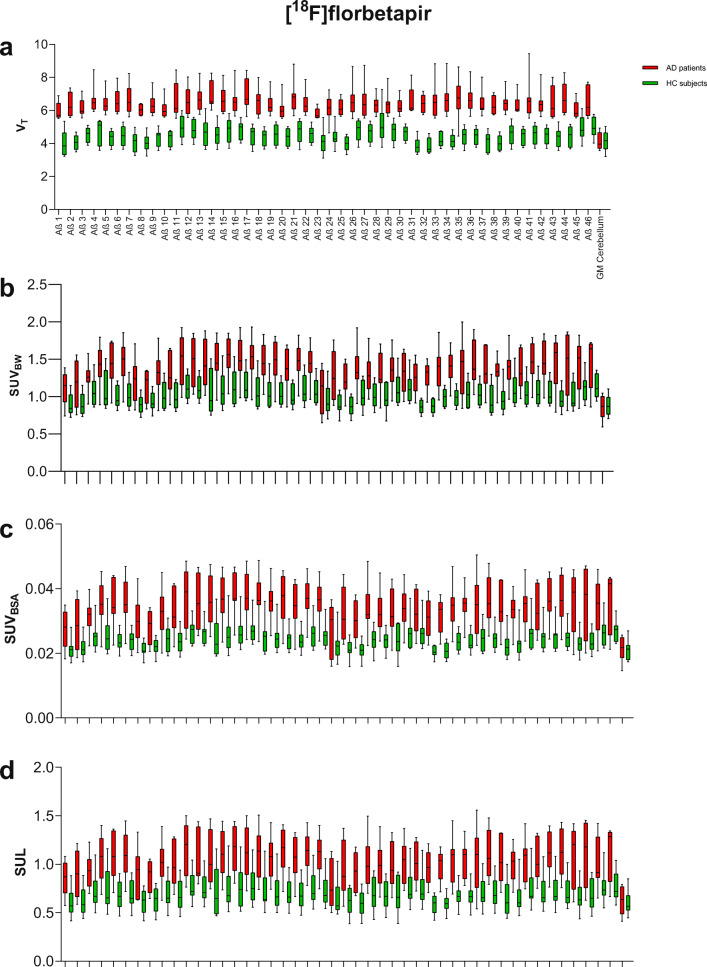
Boxplots for the SUVs and the *V*_T_ for the Aβ-specific regions (*V*_T_ higher than 8.5) and grey matter cerebellum of [^18^F]florbetapir from both AD patients (red) and controls (green). Names of the Aβ-specific regions used in this figure are illustrated in Supplementary Table 1.

All the metrics showed significant differences (*p* < 0.05) between AD patients and controls for tau- or Aβ-specific regions. Bar plots for *V*_T_ and the SUVs of [^18^F]flortaucipir and [^18^F]florbetapir in tau- or Aβ-specific regions in AD patients and controls are illustrated in Figs. 2 and 3, respectively. Clear differences in tau- or Aβ-specific regions between AD patients and HC subjects can be observed when using *V*_T_ (Figs. 2a and 3a). From Figs. 2 and 3, it can be observed that SUVs show similar variability in the grey matter cerebellum as *V*_T_ for both [^18^F]flortaucipir and [^18^F]florbetapir. No significant differences (*p* < 0.05) the in grey matter cerebellum were observed between the AD patients and controls when using the SUV metrics. Moreover, it can be seen that for previously validated reference region, both SUV and *V*_T_ are substantially lower than for other regions that show specific binding. For [^18^F]florbetapir, a SUV_BW_, SUV_BSA_, and SUL in the range between [0.64–1.18], [0.015–0.031], and [0.47–0.84], respectively, and for [^18^F]flortaucipir, a SUV_BW_, SUV_BSA_, and SUL in the range between [0.59–1.14], [0.015–0.027], and [0.38–0.86], respectively, seem to confirm absence of specific binding in the grey matter cerebellum.

**Fig. 4.**
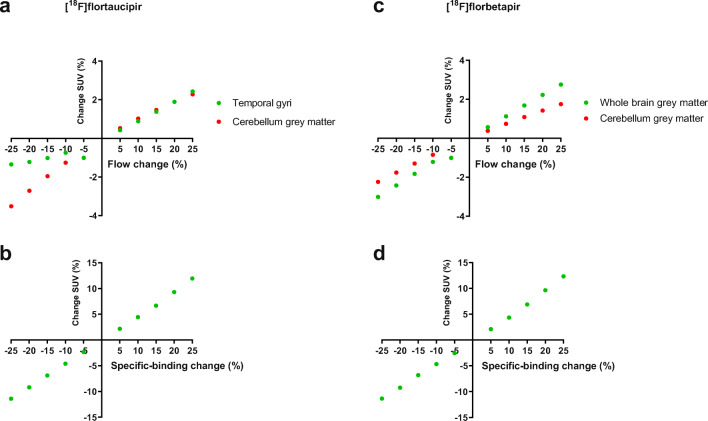
Flow and binding potential simulations illustrating the effect of **a**, **c** flow changes (± 25 %) and **b**, **d** specific binding changes (± 25 %) on the SUVs using the **a**, **b** temporal gyri ([^18^F]flortaucipir) and **c**, **d** whole-brain grey matter ([^18^F]florbetapir) as target regions, and the cerebellum grey matter as the reference region.

Simulations illustrating the effect of change in flow and specific binding on the SUVs are presented in Fig. 4. Flow simulations confirmed that the SUVs were only slightly affected (< 4 %) by changes in flow (even by flow changes of ± 25 %). In addition, the SUVs were able to detect the changes in specific binding. About 12 % increase/decrease in the SUVs was measured for a change in specific binding of ± 25 %.

## Discussion

Non-invasive SUVs can be used to verify the use of the previously validated grey matter cerebellum as the reference region in new [^18^F]flortaucipir and [^18^F]florbetapir PET scans; *i.e.* a low SUV (for [^18^F]florbetapir: SUV_BW_ ≤ 1.18, SUV_BSA_ ≤ 0.031, and SUL ≤ 0.84; and for [^18^F]flortaucipir: SUV_BW_ ≤ 1.14, SUV_BSA_ ≤ 0.027, and SUL ≤ 0.86) corresponds well with the low *V*_T_’s seen in these regions. This is particularly of importance when studies are to be conducted in case of neurodegenerative diseases or for new patient groups for which obtaining arterial input sampling is not feasible. In case of [^18^F]flortaucipir, the SUVs correlated well with *V*_T_ and were also able to discriminate between AD patients and HC subjects in tau-specific regions. The grey matter cerebellum showed similar patterns with the SUVs as with *V*_T_ for [^18^F]flortaucipir. For [^18^F]florbetapir, the SUVs correlated well with *V*_T_ for AD patients, but less for controls. SUVs did not present as clear difference as with *V*_T_ between AD patients and HC subjects in case of [^18^F]florbetapir Aβ-specific regions; however, these differences were significant. Yet, a similar trend as with *V*_T_ was observed for the grey matter cerebellum when using the SUVs.

[^18^F]florbetapir correlation between the SUVs and *V*_T_ seemed to be subject dependent. As can be seen from the scatter plots (Fig. 1) for both AD subjects and controls, different regression slopes were visualised for different subjects. This issue was predominantly observed for [^18^F]florbetapir with all SUVs. Since the AD subjects have higher *V*_T_ and SUVs, the correlations were not much affected, although for controls, the lower correlations could be due to the lower values which are more sensitive to noise. The high correlation between the [^18^F]florbetapir SUVs and the *V*_T_ shows not only that an increased SUV is observed in case of increased *V*_T_, *i.e.* increased specific binding, but also that SUVs are lower in case of decreased *V*_T_, *i.e.* absence of specific binding. A very clear delineation between AD patients and controls was not observed either when using the SUVs (Figs. 2 and 3), but significant differences were observed between the groups, and therefore, the SUVs still could be used as a surrogate to verify the use of the grey matter cerebellum as the reference region. However, the regression slopes are subject dependent and seem to have a higher impact in case of regions with higher specific binding. As can be observed from Fig. 1, a specific SUV can indicate a range of higher *V*_T_ values because of this subject-dependent regression. Therefore, the SUVs can be used as a negative indicator; *i.e.* if the SUV value of the subject is higher than the earlier specified SUV range, it clearly indicates that the grey matter cerebellum cannot be used as the reference region, although the use of SUV as a positive indicator should be interpreted with caution for [^18^F]florbetapir. Previous studies [12, 14] already validated the use of the grey matter cerebellum as the reference region for [^18^F]florbetapir quantification. It was yet unknown whether SUVs are suitable to verify the use of the grey matter cerebellum in [^18^F]florbetapir PET studies in new subjects.

In case of [^18^F]florbetapir, intra-subject differences at a regional level varied with the choice of SUV normalisation methods; therefore, each SUV was compared with the golden standard, *V*_T_. It is evident from Fig. 3 and the statistical analyses that for [^18^F]florbetapir, a similar behaviour was observed using SUVs and *V*_T_. However, differences in performance between different SUV metrics were small, and therefore, it seems that in case of [^18^F]florbetapir, any of the evaluated SUV metrics can be used to verify the grey matter cerebellum as the reference region for new [^18^F]florbetapir PET studies.

For [^18^F]flortaucipir, both subject groups showed a high correlation between the SUVs and the *V*_T_. Hence, in case of specific binding, an increased SUV and *vice versa* was observed. Some subject-dependent regression slopes were also observed in case of [^18^F]flortaucipir; however, it was relatively less evident and was almost non-existent in case of SUL. Fig. 2 illustrates a similar delineation between AD patients and controls for both the tau-specific regions and grey matter cerebellum with SUVs as was with *V*_T_. However, SUL showed the least inter-subject variation at a regional level (Fig. 1) and correlated slightly better with *V*_T_ than the other SUVs. Therefore, for [^18^F]flortaucipir, SUL may be preferred, although the improvement was not significant and therefore any of the SUVs can also be considered for the verification of use of the grey matter cerebellum as the reference region for new [^18^F]flortaucipir PET scans. For [^18^F]flortaucipir, a SUV_BW_, SUV_BSA_, and SUL in the range between [0.59–1.14], [0.015–0.027], and [0.38–0.86], respectively (Fig. 2), would confirm absence of tau binding in the grey matter cerebellum. These ranges should be warranted in a bigger patient cohort.

It may be possible that changes in cerebral blood flow and amount of specific binding induce apparent changes in SUVs. This may be problematic for reliable assessments in longitudinal PET studies. Therefore, simulations were performed to assess the effect of increase or decrease of blood flow and specific binding on the SUVs for both [^18^F]flortaucipir and [^18^F]florbetapir. Little to no blood flow impact was seen for SUVs for [^18^F]flortaucipir and [^18^F]florbetapir. This is similar to an observation in a previous study [25], where very little impact of the blood flow on [^18^F]florbetapir SUVr in AD patients was observed. SUVs were mainly influenced by the amount of specific binding but minimally by changes in blood flow for these tracers, illustrating that an increase in the amount of specific binding in the reference region can be identified based on a SUV higher than the above suggested ranges for both the tracers, which in turn suggests that the reference region for that specific scan may not be suitable. This study implicates that SUVs could be used to verify the use of the grey matter cerebellum as the reference region if the *V*_T_ is not available. Please note that this approach should be verified for each radiotracer.

## Conclusion

A low SUV, derived from 80 to 100 min p.i. and from 50 to 70 min p.i. for [^18^F]flortaucipir and [^18^F]florbetapir PET scans, respectively, seems to be a good indication for the absence of increased specific binding and thus the use of the grey matter cerebellum as the reference region for individual scans. This is of importance for subjects where a conventional way of verification (with dynamic scanning and arterial sampling) is not feasible.

## Supplementary Information

ESM 1 (DOCX 15 kb)

## Data Availability

The datasets used and/or analysed during the current study are available from the corresponding author on reasonable request.

## *Abbreviations*

SRTM: simplified reference tissue model
PET: positron emission tomography
V_T_: volume of distribution
ROIs: regions of interest
AD: Alzheimer’s disease
2T4k_V_B_: reversible two-tissue compartment model
SUV: standardised uptake value
SUV_BW_: corrected for body weight
SUV_BSA_: corrected for body surface area
SUL: corrected for lean body mass
BP_ND_: specific binding
IF: input function
Aβ: amyloid beta
IV: intravenous
TACs: time activity curves
