# Supplementary material for: Non-invasive Standardised Uptake Value for Verification of the Use of Previously Validated Reference Region for [18F]Flortaucipir and [18F]Florbetapir Brain PET Studies
Source: Mol Imaging Biol. 2021 Jan 14;23(4):550–9. doi: 10.1007/s11307-020-01572-y (PMC8277631; doi:10.1007/s11307-020-01572-y)
Supplement: Supplementary file 1 — (DOCX 15 kb) [file 11307_2020_1572_MOESM1_ESM.docx]

**Electronic Supplementary Material**

**Non-invasive Standardized Uptake Value for verification of the use of previously validated reference region for [^18^F]flortaucipir and [^18^F]florbetapir brain PET studies**

**Journal: Molecular Imaging and Biology**

Bart M. de Vries^1^, Tessa Timmers^1,2^, Emma E. Wolters^1,2^, Rik Ossenkoppele^1,2^, Sander C.J. Verfaillie^2^, Robert C. Schuit^1^, Philip Scheltens^2^, Wiesje M. van der Flier^2,3^, Albert D. Windhorst^1^, Bart N.M. van Berckel^1,2^, Ronald Boellaard^1^, Sandeep S.V. Golla^1^

*^1^Amsterdam UMC, Vrije Universiteit Amsterdam, Radiology and Nuclear Medicine,
De Boelelaan 1117, Amsterdam, Netherlands*

*^2^Amsterdam UMC, Vrije Universiteit Amsterdam, Alzheimer Center and department of Neurology,
De Boelelaan 1117, Amsterdam, Netherlands*

*^3^Amsterdam UMC, Vrije Universiteit Amsterdam, Epidemiology & Biostatistics, De Boelelaan 1117,
Amsterdam, Netherlands*

Corresponding author:

Sandeep SV Golla

Department of Radiology & Nuclear Medicine,

Amsterdam University Medical Centers, Location VUMC

De Boelelaan 1117

1081 HV Amsterdam, The Netherlands

Email: [s.golla@amsterdamumc.nl](mailto:s.golla@amsterdamumc.nl)

SUPPLEMENTARY **TABLE 1**

| **[^18^F]flortaucipir – Tau specific regions** | **[^18^F]florbetapir – Aβ specific regions** |
| --- | --- |
| 1. R Middle and inferior temporal gyri (gray) 2. L Middle and inferior temporal gyri (gray) 3. L Lateral remainder of occipital lobe (gray) 4. R Lateral remainder of occipital lobe (gray) 5. L Gyrus cinguli posterior part (gray) 6. R Gyrus cinguli posterior part (gray) 7. R Middle frontal gyrus (gray) 8. L Posterior temporal lobe (gray) 9. R Posterior temporal lobe (gray) 10. L Inferolateral remainder of parietal lobe (gray) 11. R Inferolateral remainder of parietal lobe (gray) 12. R Inferior frontal gyrus (gray) 13. L Superior parietal gyrus (gray) 14. R Superior parietal gyrus (gray) 15. R Cuneus (gray) | 1. R Anterior temporal lobe medial part (gray) 2. R Anterior temporal lobe lateral part (gray) 3. R Superior temporal gyrus (gray) 4. L Superior temporal gyrus (gray) 5. L Nucleus accumbens (gray) 6. R Nucleus accumbens (gray) 7. L Putamen (gray) 8. R Putamen (gray) 9. R Middle and inferior temporal gyri (gray) 10. L Middle and inferior temporal gyri (gray) 11. R Fusiform gyrus (gray) 12. L Fusiform gyrus (gray) 13. L Insula (gray) 14. R Insula (gray) 15. L Lateral remainder of occipital lobe (gray) 16. R Lateral remainder of occipital lobe (gray) 17. L Gyrus cinguli anterior part (gray) 18. R Gyrus cinguli anterior part (gray) 19. L Gyrus cinguli posterior part (gray) 20. R Gyrus cinguli posterior part (gray) 21. L Middle frontal gyrus (gray) 22. R Middle frontal gyrus (gray) 23. L Posterior temporal lobe (gray) 24. R Posterior temporal lobe (gray) 25. L Inferolateral remainder of parietal lobe (gray) 26. R Inferolateral remainder of parietal lobe (gray) 27. R Caudate nucleus (gray) 28. L Pallidum (gray) 29. L Precentral gyrus (gray) 30. R Precentral gyrus (gray) 31. L Gyrus rectus (gray) 32. R Gyrus rectus (gray) 33. L Orbitofrontal gyri (gray) 34. R Orbitofrontal gyri (gray) 35. L Inferior frontal gyrus (gray) 36. R Inferior frontal gyrus (gray) 37. L Superior frontal gyrus (gray) 38. R Superior frontal gyrus (gray) 39. L Postcentral gyrus (gray) 40. R Postcentral gyrus (gray) 41. L Superior parietal gyrus (gray) 42. R Superior parietal gyrus (gray) 43. L Lingual gyrus (gray) 44. R Lingual gyrus (gray) 45. L Cuneus (gray) 46. R Cuneus (gray) |
